# A desensitization protocol to ganciclovir

**DOI:** 10.1186/2045-7022-4-S3-P60

**Published:** 2014-07-18

**Authors:** Josefina Rodrigues Cernadas, Fabrícia Carolino, Ana Cláudia Carvalho, António Sarmento

**Affiliations:** 1 CH São João, Immunoallergology Department, Portugal; 2 CH São João, Infecciology Department, Portugal

## Introduction

The possibility to desensitize or induce a state of unresponsiveness to a drug that previously caused a hypersensitivity reaction is crucial when no equally safe or efficacious therapeutic alternative exists. The authors report the case of a successful desensitization to ganciclovir.

## Case report

Hospitalized HIV-infected woman, 49 years-old, with AIDS, presenting with an active cytomegalovirus (CMV) gastrointestinal infection. On the second day of therapy with intravenous ganciclovir (250 mg, q12h), the patient developed an isolated pruriginous maculopapular exanthema localized to the torso and limbs. A hypersensitivity reaction to ganciclovir was suspected; by this time, all the other comorbidities were controlled and the decision to stop the drug was taken. Because the cutaneous lesions persisted for some time although on antihistaminic and corticosteroid, an alternative treatment to CMV - foscarnet - was initiated. Biopsy of the skin lesions was performed with a positive identification of CMV. Due frequent association of foscarnet to nephrotoxicity, reintroduction of ganciclovir was attempted on the sixth day of therapy, using a modified desensitization protocol based on a previously published protocol for rapid desensitization to chemotherapy. On the first day, a cumulative dose of 125 mg was reached in 13 steps, without reactions. On the next day, adding two steps to the protocol, a dose of 250mg was safely administered. Dose increments between steps were relatively small, in an attempt to minimize the risk of reaction. The association of foscarnet and 250mg of ganciclovir was maintained for four days. The total daily therapeutic dose (500mg) of ganciclovir was reached under supervised slow perfusion (over 90 minutes) of 250mg every 12 hours. No clinical or analytical reactions were reported so foscarnet could be suspended, keeping intravenous ganciclovir as monotherapy. After two weeks under full intravenous dose, the switch to oral treatment with valganciclovir was well tolerated.

## Comments

To our knowledge, there are no published protocols of desensitization to ganciclovir. As viral infections might increase the risk of hypersensitivity reactions, treatment with foscarnet was maintained until the therapeutic dose of ganciclovir was reached, assuring full CMV treatment along all the process.

